# Exploring the Visual Profile in Pediatric Scoliosis: Insights from a Controlled Comparative Study

**DOI:** 10.22336/rjo.2026.30

**Published:** 2026

**Authors:** Iulian Manac, Sanda Jurja, Nicoleta Leopa, Florina Anca Manac, Traian Virgiliu Surdu, Monica Surdu, Ioana Georgia Oglindă, Alexandru Vicențiu Vâlcu, Stere Popescu, Florin Daniel Enache

**Affiliations:** 1 Ovidius University, Faculty of Medicine, Constanţa, Romania; 2 Department of Pediatric Surgery, Emergency Hospital of Constanța, Constanța, Romania; 3 Department of Ophthalmology Anesthesia and Intensive Care Unit, Emergency Hospital of Constanța, Constanța, Romania; 4 Department of General Surgery, Emergency Hospital of Constanța, Constanța, Romania; 5 Department of Anesthesia and Intensive Care Unit, Emergency Hospital of Constanța, Constanța, Romania; 6 Department of Recovery, Physical Medicine and Balneology, Recovery Sanatorium, Techirghiol, Constanţa, Romania; 7 Department of Neonatology, Emergency Hospital of Constanța, Constanța, Romania; 8 Department of Orthopedics, Emergency Hospital of Constanța, Constanța, Romania

**Keywords:** visual disturbances, idiopathic scoliosis, pediatric population, calcium and magnesium, postural control, BMI = Body Mass Index

## Abstract

**Background:**

Idiopathic scoliosis is a complex spinal deformity that often emerges during growth and has a multifactorial etiology. Recent evidence suggests that visual disturbances may affect postural alignment and potentially influence spinal curvature patterns. However, the relationship between visual impairments and scoliosis, especially S-shaped curves, remains insufficiently understood. The aim was to investigate the association between visual disturbances and spinal curvature characteristics in pediatric patients with double major idiopathic scoliosis, and to explore the correlation between visual status and selected metabolic biomarkers.

**Materials and methods:**

This retrospective controlled study included 73 pediatric patients under the age of 16, diagnosed with double major idiopathic scoliosis between January 2020 and 2025. Patients were divided into two groups: 38 with visual disturbances (myopia, hypermetropia, astigmatism, strabismus, or partial blindness) and 35 without visual impairments (control group). Radiographic parameters, clinical characteristics, and biochemical markers were analyzed using univariate and multivariate statistical methods.

**Results:**

Patients with visual disturbances exhibited a significantly more cranial thoracic apical vertebra level (T6 vs. T8, p < 0.01), greater thoracic Cobb angles, and lower mean serum calcium and magnesium levels compared with the control group (*p* < 0.05). Multinomial logistic regression showed that hypomagnesemia and earlier thoracic apical vertebra level were independently associated with visual disturbances.

**Discussion:**

The present study provides preliminary evidence that visual disturbances may be associated with specific spinal and metabolic characteristics in children with idiopathic double-curve scoliosis. Although causal relationships cannot be established, these findings support further investigation of visual assessment as a potential component of multidisciplinary scoliosis evaluation and early screening strategies.

**Conclusions:**

Visual impairments in pediatric patients with double major idiopathic scoliosis are associated with altered spinal morphology and lower calcium-magnesium levels. These findings support the potential role of visual and metabolic screening in the comprehensive assessment and management of scoliosis.

## Introduction

Idiopathic scoliosis is the most prevalent structural spinal deformity in children, characterized by a lateral curvature of the spine exceeding a 10° Cobb angle and the absence of vertebral abnormalities or neuromuscular diseases [1]. The precise etiology remains unclear, but prevailing ideas suggest a complex origin involving genetic predisposition, impaired proprioception, vestibular dysfunction, hormonal factors, and anomalies in postural regulation [2]. Among these, sensory system disturbances—including those affecting vision—have gained growing attention as potential contributors to the initiation or progression of spinal curvatures [3,4].

The function of the visual system in postural regulation is thoroughly established [5]. The central nervous system sustains spatial orientation, balance, and musculoskeletal alignment by continuously integrating visual input with vestibular and somatosensory signals [6]. In children and adolescents, whose neurosensory integration is still developing, visual impairments such as myopia, hypermetropia, astigmatism, strabismus, or partial visual deprivation may result in compensatory mechanisms, including asymmetric head positioning, body tilting driven by ocular dominance, or impaired trunk coordination [7]. These alterations in postural strategy could, over time, affect spinal growth and the progression of curvature [8].

The concept that idiopathic scoliosis, especially double major idiopathic scoliosis, may be affected by impaired visual input is substantiated by many biomechanical factors [9]. Double-major idiopathic scoliosis features opposing thoracic and lumbar curvatures, necessitating intricate compensatory alignment to preserve overall sagittal and coronal equilibrium [1]. Visual feedback is essential in modulating these compensatory adjustments. Any deficit in binocular vision, depth perception, or oculomotor control may disrupt this delicate balance, perhaps encouraging the development of more complex scoliotic patterns [4,10]. Moreover, asymmetric visual dominance or ocular fixation disorders may be mirrored by corresponding axial deviations or muscle activation imbalances in the trunk [11].

Beyond biomechanical interactions, recent studies have explored potential biochemical correlates between scoliosis and visual function, focusing on micronutrients essential to neuromuscular and retinal health [9,10]. Calcium and magnesium are involved in synaptic transmission, regulation of muscle tone, and retinal cell signaling [12]. Deficiencies in these minerals have been associated with both postural instability and visual system impairment, implying a common metabolic pathway that influences both disorders [13]. Despite these theoretical links, few clinical studies have investigated the direct relationship between visual problems and idiopathic scoliosis, and even fewer have looked at curve morphology or biochemical markers as potential mediators. Most published papers are limited to case series or uncontrolled designs, or focus solely on neuro-ophthalmologic findings without spinal examination [14,15].

This study aimed to investigate the association between visual impairments and spinal characteristics—particularly thoracic and lumbar apical vertebra level, Cobb angles, and axial rotation—in pediatric patients with double major idiopathic scoliosis. In addition, we assessed the potential role of serum calcium and magnesium levels in modulating this relationship, using a controlled comparative approach.

## Materials and methods

### Study group

This was a retrospective, controlled, observational study conducted between January 2020 and January 2025, aimed at investigating the association between visual disturbances and spinal and biochemical characteristics in pediatric patients diagnosed with double major idiopathic scoliosis. All patients were consecutively selected from the hospital’s pediatric orthopedic records. Only cases with complete datasets for all study variables were included, and patients with missing values were excluded from the analysis. A total of 96 patient records were initially screened. After applying the inclusion and exclusion criteria, 73 patients were eligible for analysis. All included patients were diagnosed with double major idiopathic scoliosis, confirmed through standard radiological evaluation. Only patients with double major idiopathic scoliosis were selected to reduce anatomical variability and ensure biomechanical consistency across the study cohort. This curve pattern, involving both thoracic and lumbar segments, allows for more symmetrical postural compensation compared to single-curve scoliosis. Its inclusion facilitated a more controlled investigation of the potential interactions between spinal morphology, visual disturbances, and serum calcium/magnesium levels.

The study population was divided into two groups: Group I consisted of 38 patients with idiopathic scoliosis and documented visual disturbances, including myopia, hypermetropia, astigmatism, strabismus, or partial blindness, as confirmed by ophthalmologic evaluation; Group II (control group) included 35 patients with idiopathic double major scoliosis but without any visual impairments. Control group patients were selected from the same clinical period and matched to the study group on age, body mass index (BMI), and residential background (urban vs. rural) to reduce selection bias and ensure comparability between groups. Frequency matching was used to ensure similar distributions between the study and control groups. Patients in the control group were selected from the same five-year period and were matched at the group level, rather than on a one-to-one basis.

**Fig. 1:**
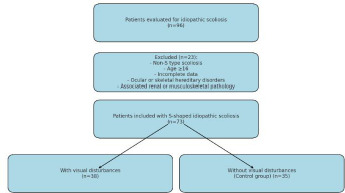
Flowchart of patient selection and study grouping

Inclusion criteria were: 1) age under 16 years; 2) confirmed diagnosis of double major idiopathic scoliosis; 3) availability of complete clinical, biochemical (serum calcium and magnesium), and visual assessment data. For the control group: absence of visual pathology as confirmed by normal ophthalmologic screening. Exclusion criteria included: 1) congenital, neuromuscular, or syndromic scoliosis; 2) coexisting osteoarticular or renal conditions; 3) prior orthopedic treatment for spinal deformities; 4) hereditary ocular or skeletal disorders. A Flow chart is available (Fig. 1).

All patients were evaluated using standardized imaging and laboratory protocols. Data were collected from electronic medical records, anonymized before analysis, and handled in compliance with institutional ethical standards.

### Data Collection

Data were collected retrospectively from electronic medical records and standardized clinical documentation forms completed during patient evaluation. All patients included in the study underwent complete orthopedic, biochemical, and ophthalmologic assessments as part of their routine clinical management.

The following demographic variables were recorded: age (in years), gender, environment of origin (urban or rural), and BMI (kg/m^2^). Radiographic parameters were extracted from standardized anteroposterior and lateral spine radiographs. The following variables were assessed: type of scoliotic curvature, with a focus on double major idiopathic scoliosis, curve direction: thoracic dextroconvex + lumbar levoconvex, or thoracic levoconvex + lumbar dextroconvex, presence of axial vertebral rotation, as determined by radiographic analysis, thoracic apical vertebra level: vertebral level marking the apex of thoracic curvature, lumbar apical vertebra level: vertebral level marking the apex of lumbar curvature, thoracic Cobb angle and lumbar Cobb angle, measured using the standard Cobb method.

### Study Endpoints

The primary endpoint of the study was to assess the association between visual disturbances and spinal curvature characteristics in pediatric patients with double major idiopathic scoliosis. Specifically, we aimed to evaluate whether the presence of visual impairments was associated with differences in thoracic and lumbar apical vertebra levels, Cobb angles, and curve direction.

The secondary endpoint was to investigate the relationship between serum calcium and magnesium levels and the presence and type of visual disturbances in the study population.

### Ethical consideration

In compliance with the Declaration of Helsinki on human testing, the study was authorized by the Local Ethics Commission for the Approval of Clinical and Research Developmental Studies (approval no. 23/01.12.2020). For all participants, informed consent to participate was obtained from their parents or legal guardians at the time of hospital admission.

### Statistical analysis

Statistical analysis was performed using SPSS (V28) and GraphPad Prism 10. Continuous variables were reported as mean ± standard deviation, and categorical variables were expressed as frequencies with percentages. Group comparisons were performed using the χ2 test or the Mann-Whitney U test. A p-value < 0.05 was considered statistically significant. Missing data were handled through case-wise deletion; only complete records were analyzed to preserve data integrity. A p-value < 0.05 was considered statistically significant.

## Results

At baseline, the two study groups—patients with visual impairments (n = 38) and the control group without visual impairments (n = 35)—were comparable in terms of demographic and clinical characteristics (Table 1). The mean age was virtually identical between groups, 13.42 ± 1.57 years in the visual impairment group and 13.26 ± 1.76 years in the control group (*p* = 0.985). Most patients originated from urban areas in both cohorts (92.1% vs. 80%), with no statistically significant difference in environmental distribution (*p* = 0.129). Gender distribution also showed no significant variation, with 55.3% males in the visual impairment group and 37.1% in the control group (*p* = 0.120). Body mass index values were similar: 23.92 ± 5.09 kg/m^2^ in the visual impairment group compared to 23.25 ± 5.8 kg/m^2^ in controls (*p* = 0.596).

**Table 1:** Baseline characteristics of pediatric scoliosis patients with and without visual impairments

Variables	Visual Impairments Group (n=38)	Control Group (n=35)	p-value
Age (years)*	13.42 ± 1.57	13.26 ± 1.76	0.985
Environment Urban Rural	35 (92.1) 3 (7.9)	28 (80) 7 (20)	0.129
Gender Male Female	21 (55.3) 17 (44.7)	13 (37.1) 22 (62.9)	0.120
Body Mass Index (kg/m^2^)*	23.92 ± 5.09	23.25 ± 5.8	0.596
Types of curvatures Thoracic dextroconvex, lumbar levoconvex Thoracic levoconvex, lumbar dextroconvex	29 (76.3) 9 (23.7)	26 (74.3) 9 (25.7)	0.841 0.542
Axial vertebral rotation	15 (39.5)	19 (54.3)	0.204
Cobb angle * Thoracic Lumbar	24.47 ± 10.2 15.21 ± 5.35	22.77 ± 8.92 15.14 ± 6.09	0.258 0.454

With percentages in parentheses unless indicated otherwise; *Values are mean (standard deviation)

Curve pattern types were evenly distributed across groups. A thoracic dextroconvex and lumbar levoconvex configuration was observed in 76.3% of visually impaired patients and 74.3% of controls (*p* = 0.841), while the reverse configuration (thoracic levoconvex, lumbar dextroconvex) occurred in 23.7% and 25.7%, respectively (*p* = 0.542). Axial vertebral rotation was present in 39.5% of the visual impairment group and 54.3% of controls (*p* = 0.204). Regarding spinal deformity severity, the thoracic Cobb angle averaged 24.47 ± 10.2° in the visual impairment group, compared to 22.77 ± 8.92° in controls (*p* = 0.258). Lumbar Cobb angles were also similar, with mean values of 15.21 ± 5.35° and 15.14 ± 6.09°, respectively (*p* = 0.454).

**Fig. 2:**
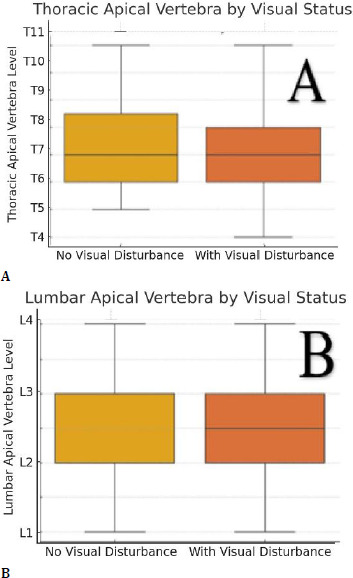
Thoracic and lumbar apical vertebra level in pediatric scoliosis according to visual status. (A) Boxplot showing the distribution of thoracic apical vertebra level; (B) Boxplot representing lumbar apical vertebra level

The analysis of the apical vertebral level along the spine (Fig. 2) revealed subtle yet relevant differences between scoliosis patients with and without visual disturbances. At the thoracic level, the apical vertebra level—the vertebral apex of curvature—was located slightly more cranially in patients with visual impairments, with a median value tending toward higher thoracic vertebrae compared to their visually intact counterparts. This anterior displacement may reflect a shift in postural compensation mechanisms in response to altered visual input, potentially influencing the biomechanical balance of the thoracic spine. The thoracic apical vertebra tended to be lower in patients with visual disturbances (median T6 vs. T7 in controls), suggesting a more proximal inflection point.

In contrast, the lumbar apical vertebra level demonstrated a more homogeneous distribution across both groups. The median vertebral level and interquartile range remained largely similar regardless of visual status, suggesting that the lumbar spine is either less sensitive to sensory compensatory input or more stable in its axial positioning during scoliosis progression. Taken together, these findings suggested that visual disturbances might have a more pronounced impact on upper spinal curvature dynamics, possibly due to the thoracic spine’s greater involvement in maintaining postural alignment in response to impaired visual cues.

**Fig. 3:**
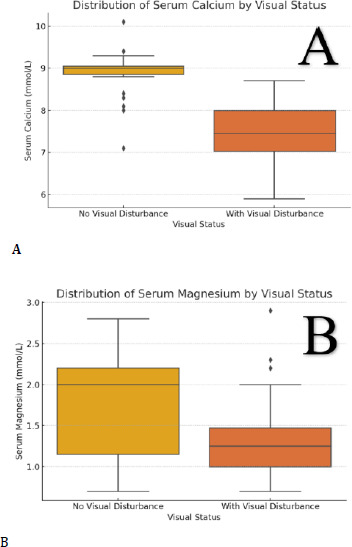
Distribution of serum calcium and magnesium in pediatric scoliosis patients according to visual status. (A) Boxplot showing the distribution of serum calcium levels; (B) Boxplot illustrating the distribution of serum magnesium levels

The comparative boxplots of serum calcium and magnesium levels revealed distinct patterns between pediatric scoliosis patients with and without visual disturbances (Fig. 3). Patients with visual disturbances had consistently lower serum calcium levels than those without visual disturbances. The distribution was not only shifted downward but also displayed less variability, indicating a more uniform hypocalcemic profile within the visually impaired group. This trend suggested that calcium deficiency might be more prevalent or more pronounced among scoliosis patients with concurrent visual system involvement. A similar pattern was observed for serum magnesium levels. Although the interquartile range was broader, the median magnesium value was lower in the group with visual disturbances. This observation reinforced the hypothesis that magnesium, like calcium, might contribute to the pathophysiology of visual comorbidities in scoliosis.

Table 2 presents the results of a multivariate logistic regression assessing the association between several clinical and demographic variables and the presence of visual impairments in pediatric scoliosis patients. Among the predictors analyzed, serum calcium and magnesium levels were the only variables significantly associated with visual disturbances. Lower calcium levels were strongly predictive, with a regression coefficient of –5.585 (*p* = 0.001) and an odds ratio (OR) of 0.004 [95% CI: 0.000–0.088], indicating that hypocalcemia was associated with a substantially increased likelihood of visual impairment. Similarly, lower magnesium levels were also significantly associated with visual deficits, with a coefficient of –2.961 (*p* = 0.018) and an OR of 0.052 [95% CI: 0.004–0.603].

**Table 2:** Factors associated with visual impairments in multivariate analysis

Variable	Myopia		Strabismus		Hypermetropia		Astigmatism	
	OR [95% CI]	*p-value	OR [95% CI]	*p- value	OR[95% CI]	*p-value	OR [95% CI]	*p- value
Serum Calcium	0.002 [ 0.000-0.351]	*0.001*	0.286 [0.012-6.960]	*<0.001*	0.001 [0.000-0.063]	*0.001*	0.001 [0.000-0.068]	*0.001*
Serum Magnesium	0.013 [0.000-0.351]	*0.010*	0.006 [0.000-0.316]	*0.012*	0.063 [0.003-1.407]	0.081	0.002 [0.000-0.122]	*0.002*

Values in italics indicate statistical significance (P < 0.050). B: unstandardized coefficient; S.E.: standard error; OR, odds ratio; CI, confidence interval

Other variables, including gender (OR = 0.703; *p* = 0.756), environment (urban vs. rural, OR = 0.041; *p* = 0.074), curve pattern (thoracic dextroconvex/lumbar levoconvex, OR = 1.459; *p* = 0.776), and axial vertebral rotation (OR = 1.951; *p* = 0.572), did not show statistically significant associations. These findings suggested that biochemical imbalances—particularly involving calcium and magnesium—might play a more crucial role in the development or presence of visual disturbances than demographic or postural factors. The strong inverse associations identified reinforce the potential need for routine metabolic screening and correction in pediatric patients with scoliosis and coexisting visual abnormalities.

The results of the multinomial logistic regression (Table 3) highlighted the differential influence of serum calcium and magnesium on specific visual disturbances among scoliosis patients. Hypocalcemia was significantly associated with an increased risk of all four visual conditions. The odds of presenting with myopia were markedly elevated with lower calcium levels (OR = 0.002, 95% CI: [0.000–0.351], p = 0.001). Similarly, calcium levels were strongly inversely associated with strabismus (OR = 0.286, 95% CI: [0.012–6.960], *p* < 0.001), hypermetropia (OR = 0.001, 95% CI: [0.000–0.063], *p* = 0.001), and astigmatism (OR = 0.001, 95% CI: [0.000–0.068], *p* = 0.001). These findings underscore a consistent pattern: reduced serum calcium levels are associated with a significantly increased risk of refractive and ocular alignment disorders.

**Table 3:** Multivariable associations between metabolic markers and specific types of visual impairment

Variables	B	S.E.	p value	OR [95% CI]
Gender	-0.352	1.133	0.756	0.703 [0.076-6.483]
Environment	-3.192	1.786	0.074	0.041 [0.001-1.361]
Thoracic dextroconvex, lumbar levoconvex	0.378	1.330	0.776	1.459 [0.108-19.771]
Axial vertebral rotation	0.668	1.182	0.572	1.951 [0.192-19.771]
Serum Calcium	-5.585	1.610	*0.001*	0.004 [0.000-0.088]
Serum Magnesium	-2.961	1.253	*0.018*	0.052 [0.004-0.603]
Constant	0.082	0.234	0.000	1.086

OR = odds ratio; CI = confidence interval; *MVA = multivariate analysis; Values in italics indicate statistical significance (P < 0.005)

Magnesium levels were also significantly associated with visual disturbances, although to a slightly lesser extent. Statistically significant associations were observed with myopia (OR = 0.013, 95% CI: [0.000–0.351], *p* = 0.010), strabismus (OR = 0.006, 95% CI: [0.000–0.316], *p* = 0.012), and astigmatism (OR = 0.002, 95% CI: [0.000–0.122], *p* = 0.002). The association with hypermetropia did not reach statistical significance (*p* = 0.081), although the OR remained below 1, suggesting a potential trend.

## Discussions

Idiopathic scoliosis in children and adolescents is a complex three-dimensional deformity of the spine, whose pathogenesis is thought to involve an interplay of genetic, biomechanical, neuromuscular, and environmental factors [4-7]. While traditionally studied in isolation, growing evidence suggests that scoliosis may co-occur with sensory and metabolic abnormalities, raising the possibility of broader systemic involvement [11-14].

The present study offers a novel perspective on the intersection between spinal deformities and visual disturbances in pediatric patients with idiopathic scoliosis. By conducting a comparative analysis between children with and without visual impairments, the study explored whether visual input deficits might be associated with differences in spinal curvature patterns, apical vertebra level, and biochemical markers—specifically serum calcium and magnesium. Although the association between calcium levels and visual disturbances was statistically significant, the extremely low odds ratio (OR = 0.004; 95% CI: 0.000–0.088) suggested potential model overfitting or sensitivity to outliers. This finding should therefore be interpreted with caution, especially considering the limited sample size. Larger studies are needed to validate the strength and consistency of this association.

Although the baseline analysis revealed no significant differences in general anthropometric parameters or curvature types between the two groups, subtle but important biomechanical distinctions emerged. Notably, patients with visual disturbances demonstrated a cranial shift in the thoracic inflection point, which might reflect altered postural control mechanisms. This finding aligns with prior studies that suggest visual input is integral to postural alignment and proprioceptive feedback in children [16]. In visually impaired individuals, compensatory strategies such as increased truncal rigidity or asymmetric weight distribution have been described, potentially altering the progression of spinal curvature.

The lack of difference in the lumbar inflection point between groups suggested that lower spinal segments might be less susceptible to visual sensory modulation. This observation might relate to the thoracic spine’s greater involvement in postural adaptation and its anatomical proximity to the vestibulo-ocular system, which is critical for head and neck control. Although patients with visual disturbances tended to have a lower thoracic apical vertebra (median T6 vs. T7 in controls), this difference did not reach statistical significance in all analyses. As such, these findings should be interpreted with caution and considered hypothesis-generating rather than conclusive. Larger studies with more detailed imaging and biomechanical modeling are needed to explore potential associations between visual input and thoracic curvature morphology.

One of the most striking findings of the present study is the consistent association between hypocalcemia and hypomagnesemia and the presence of visual impairments in pediatric scoliosis. In both the general logistic regression model and the multinomial analysis stratified by visual impairment type, low serum calcium levels were robustly associated with myopia, strabismus, hypermetropia, and astigmatism. Similarly, serum magnesium was independently associated with myopia, strabismus, and astigmatism. These findings are supported by existing evidence that links calcium and magnesium dysregulation to neuro-ophthalmologic dysfunction [17,18]. Calcium ions play a pivotal role in phototransduction, synaptic signaling, and retinal neurotransmission [19].

Hypocalcemia has been implicated in retinal dystrophies, impaired lens accommodation, and delayed visual-motor integration in children. Magnesium, on the other hand, is essential for retinal ganglion cell metabolism, intraocular pressure regulation, and neurovascular coupling. This association highlighted a potential relationship between calcium and magnesium deficiencies and visual disturbances in patients with idiopathic scoliosis. However, given the cross-sectional and retrospective design of our study, no causal inference could be made. It is also possible that these biochemical alterations were epiphenomena rather than direct contributors. Further prospective and mechanistic studies are needed to explore the direction and nature of this association.

Experimental studies have shown that magnesium deficiency can lead to optic nerve vulnerability, particularly under conditions of oxidative stress or altered cerebrovascular tone [20,21]. Notably, the independent association between these electrolyte abnormalities and visual disturbances was more significant than any postural or demographic variable, including gender, environment, or Cobb angle. This reinforces the hypothesis that mineral homeostasis may represent a modifiable risk factor in the co-expression of scoliosis and visual dysfunction.

These findings carry important clinical implications. First, routine biochemical screening of serum calcium and magnesium could be considered at the time of diagnosis, especially in pediatric scoliosis patients presenting with coexisting refractive errors or ocular alignment abnormalities. This early assessment might help identify patients who could benefit from timely nutritional intervention. Second, interventional studies assessing the effect of mineral supplementation on both postural and visual outcomes could be valuable, especially given the cost-effectiveness and safety profile of such therapies.

*Strengths and Limitations*

Despite its strengths—including a controlled comparative design and comprehensive statistical modeling—this study had limitations. The relatively small sample size might limit the generalizability of results. A power analysis indicated that a minimum of 64 patients would be required to detect medium effect sizes, and our final sample of 73 patients met this criterion. Nonetheless, the relatively small number of patients with coexisting visual disturbances and idiopathic S-shaped scoliosis might limit the statistical power of subgroup analyses. Moreover, most scoliosis cases included in the study were mild to moderate in severity, reflecting typical patterns observed in early pediatric assessments. Although the total number of included patients met the threshold established by our initial power analysis, further subgroup analyses (based on type of visual impairment or biochemical markers) might be underpowered. This limitation affected the robustness of subgroup comparisons and should be addressed in future prospective studies with larger populations.

In addition, although we controlled for major confounders, residual bias from genetic, behavioral, or environmental factors could not be ruled out. No formal correction for multiple comparisons was applied due to the exploratory design and limited sample size. As a result, some statistically significant associations might be due to chance, and the findings should be interpreted with caution until replicated in larger, hypothesis-driven studies. Longitudinal studies are needed to determine causality and temporal relationships between metabolic imbalance and visual-spinal comorbidity. Furthermore, data on dietary intake, socioeconomic background, or comorbidities that could influence mineral metabolism were not collected. These unmeasured confounders might partially account for the observed differences in calcium and magnesium levels, and future studies should include these parameters to refine the interpretation of metabolic findings.

## Conclusions

In summary, visual disturbances in pediatric scoliosis are associated with altered thoracic curve positioning and significant reductions in serum calcium and magnesium levels. These findings underscore the potential role of metabolic screening in the early identification and multidisciplinary management of affected patients.
